# Predicting Pain in People With Sickle Cell Disease in the Day Hospital Using the Commercial Wearable Apple Watch: Feasibility Study

**DOI:** 10.2196/45355

**Published:** 2023-03-14

**Authors:** Rebecca Sofia Stojancic, Arvind Subramaniam, Caroline Vuong, Kumar Utkarsh, Nuran Golbasi, Olivia Fernandez, Nirmish Shah

**Affiliations:** 1 Duke Sickle Cell Comprehensive Care Unit Department of Medicine, Division of Hematology Duke University Hospital Durham, NC United States; 2 Brody School of Medicine East Carolina University Greenville, NC United States; 3 Department of Pediatric Hematology Amsterdam University Medical Centers University of Amsterdam Amsterdam Netherlands; 4 Department of Engineering Sciences and Applied Mathematics Northwestern University Evanston, IL United States; 5 Joan & Sanford I Weill Medical College Cornell University New York, NY United States; 6 University of North Carolina at Chapel Hill Chapel Hill, NC United States

**Keywords:** sickle cell disease, vaso-occlusive crises, mobile health, consumer wearable, Apple Watch, machine learning, pain, prediction, smartwatch, wearable, predict

## Abstract

**Background:**

Sickle cell disease (SCD) is a genetic red blood cell disorder associated with severe complications including chronic anemia, stroke, and vaso-occlusive crises (VOCs). VOCs are unpredictable, difficult to treat, and the leading cause of hospitalization. Recent efforts have focused on the use of mobile health technology to develop algorithms to predict pain in people with sickle cell disease. Combining the data collection abilities of a consumer wearable, such as the Apple Watch, and machine learning techniques may help us better understand the pain experience and find trends to predict pain from VOCs.

**Objective:**

The aim of this study is to (1) determine the feasibility of using the Apple Watch to predict the pain scores in people with sickle cell disease admitted to the Duke University SCD Day Hospital, referred to as the Day Hospital, and (2) build and evaluate machine learning algorithms to predict the pain scores of VOCs with the Apple Watch.

**Methods:**

Following approval of the institutional review board, patients with sickle cell disease, older than 18 years, and admitted to Day Hospital for a VOC between July 2021 and September 2021 were approached to participate in the study. Participants were provided with an Apple Watch Series 3, which is to be worn for the duration of their visit. Data collected from the Apple Watch included heart rate, heart rate variability (calculated), and calories. Pain scores and vital signs were collected from the electronic medical record. Data were analyzed using 3 different machine learning models: multinomial logistic regression, gradient boosting, and random forest, and 2 null models, to assess the accuracy of pain scores. The evaluation metrics considered were accuracy (*F*_1_-score), area under the receiving operating characteristic curve, and root-mean-square error (RMSE).

**Results:**

We enrolled 20 patients with sickle cell disease, all of whom identified as Black or African American and consisted of 12 (60%) females and 8 (40%) males. There were 14 individuals diagnosed with hemoglobin type SS (70%). The median age of the population was 35.5 (IQR 30-41) years. The median time each individual spent wearing the Apple Watch was 2 hours and 17 minutes and a total of 15,683 data points were collected across the population. All models outperformed the null models, and the best-performing model was the random forest model, which was able to predict the pain scores with an accuracy of 84.5%, and a RMSE of 0.84.

**Conclusions:**

The strong performance of the model in all metrics validates feasibility and the ability to use data collected from a noninvasive device, the Apple Watch, to predict the pain scores during VOCs. It is a novel and feasible approach and presents a low-cost method that could benefit clinicians and individuals with sickle cell disease in the treatment of VOCs.

## Introduction

Sickle cell disease (SCD) is an inherited monogenic disorder that affects millions of individuals across the world and is estimated to affect 300,000 new children every year [1-3]. The sickled red blood cells have adhesive properties to other cells, building up in blood vessels and blocking blood flow to tissues. This process is known as vaso-occlusive crises (VOCs) and leads to the onset of a complex cascade of vaso-occlusion, inflammation, and ischemia, ultimately resulting in complications such as acute pain [4]. VOCs are often referred to simply as “pain crises” and frequently do not have a specific cause [5]. Shah et al looked at over 8000 individuals with sickle cell disease over a 3-year period and reported that each patient averaged 3.3 VOCs per year [6].

VOCs are associated with a decreased health-related quality of life and are a significant morbidity for individuals with sickle cell disease; it is the most common cause of hospitalization in SCD [5,7]. The treatment for VOCs is currently limited to analgesics such as opioids, which are given in proportion to the reported level of pain [8]. Although most patients manage their pain at home, if VOCs cannot be controlled, hospitalization is required to administer intravenous analgesics and fluids. Ultimately, due to the frequency and unpredictability of VOCs, there are high health care usage and costs for patients with sickle cell disease [9]. Having an ability to objectively determine the timing and intensity of VOCs may improve pain management and lead to an increased health-related quality of life as well as lower resource usage in patients living with sickle cell disease.

Recent efforts to better understand pain include using machine learning techniques to help analyze pain and associated physiological data. Machine learning is the usage of data and analytics to predict outcomes, allowing computers to execute operations without explicit instructions. Machine learning models have been applied to SCD and non-SCD pain–related research to visualize how pain indicators relate to subjective pain [10-12]. Health care studies involving machine learning have evaluated patients suffering from chronic and postoperative pain using heart rate variability (HRV), brain activity, and clinical data to create complex multivariable models that attempt to predict pain levels [10]. In using the mentioned variables, researchers built models that successfully predicted self-reported pain intensity or postoperative pain intensity, but the methods used to collect data were expensive and difficult to perform on a large scale.

Even as this research continues, a gap exists in the usage of sustainable, cost-effective methods to better understand pain in SCD. We believe that consumer wearable devices, in particular, smartwatches, may be a way to fill that gap. Consumer wearable devices are increasing in popularity globally and can be an affordable way to gather large amounts of continuous and real-time biometric data both in and out of a clinical setting [13,14]. Biometric data collected by consumer wearables can include heart rate (HR), HRV, step count, burned calories, and oxygen saturation. Our research team previously evaluated data collected from the consumer wearable Microsoft Band 2 to assess if subjective pain scores could be predicted in individuals living with sickle cell disease. The study was able to predict subjective pain scores using the data collected from Microsoft Band 2, pain score data, and a regression machine learning model, with a correlation of 0.706 in adult patients during their time in the Day Hospital for a painful VOC [6]. The Microsoft Band 2 provided robust data but had a limited battery life of approximately 6 hours and has since been discontinued. To continue our research into the feasibility of using consumer wearables as a sustainable and cost-effective method to better understand pain, we adopted the Apple Watch based on its popularity and global acceptance, as well as the robust data collection of Apple Health Kit and the open access to raw collected data. This study looked to evaluate the performance of various machine learning models on data collected from the Apple Watch to predict reported pain scores in individuals with sickle cell disease suffering from VOC.

The aim of this study is to (1) determine the feasibility of using the Apple Watch to predict the pain scores in people with sickle cell disease admitted to the Day Hospital and (2) build and evaluate machine learning algorithms to predict the pain scores of VOCs with the Apple Watch.

## Methods

### Data Collection

Following approval from the Duke Institutional Review Board, patients meeting the inclusion criteria and entering the Day Hospital with a VOC between July 2021 and September 2021 were approached and consented. Patients included had to have a confirmed SCD diagnosis, 18 years of age or older, and admitted with a primary diagnosis of VOC. The study team provided participants with an Apple Watch Series 3 to be worn for the duration of their visit. The Apple Watch was attached to the wrist of the participant and placed in “Other” exercise mode. Exercise mode allowed for more continuous collection of HR and other Apple Health Kit data, collecting HR data every 5 seconds [15]. This allowed us to retrieve the maximum amount of HR data the Apple Watch could record during the time the participants were enrolled in the study. Biometric data collected via the Apple Watch included HR, active calorie burn, and basal calories burned. Pain scores and vital sign variables including blood pressure, pulse, and temperature, as well as demographics including age, SCD genotype, sex, and ethnicity were extracted from the electronic medical records (EMRs). Patients completed the study either due to discharge following pain management, the closing of the Day Hospital, or transferring to the emergency room. Data from the Apple Watch were extracted from Apple Health Kit and exported as XML files, converted to XLSX, and analyzed using Python (version 3.9.6; Python Software Foundation).

### Outcomes

Pain scores, HR data, calculated HRV, and calories burned were combined using the minimum absolute time difference between time stamps, to create a cohesive data set. We assumed the pain score to remain the same for up to ±15 minutes when each pain score was recorded, in order to expand the usable data set.

We expanded our data set by self-calculating HRV from the HR data, based on existing evidence of HRV’s relationship to pain [16]. Classically, HRV is calculated by analyzing the electrocardiography data. Due to the lack of electrocardiography data, we instead used the fluctuations in the HR to calculate HRV, which can also be called pulse rate variability. Previous studies have shown that pulse rate variability and HRV are significantly correlated, and the values are very close to each other for measurements [17]. There are multiple metrics to represent HRV [18], and we chose the root-mean-square of successive differences between normal heartbeats of 70 and 110 beats per minute. The time difference between successive normal heartbeats was noted for 5 minutes, the values of their successive differences were calculated and squared, and then the result was then averaged and squared off.

### Analysis

**Figure 1:**
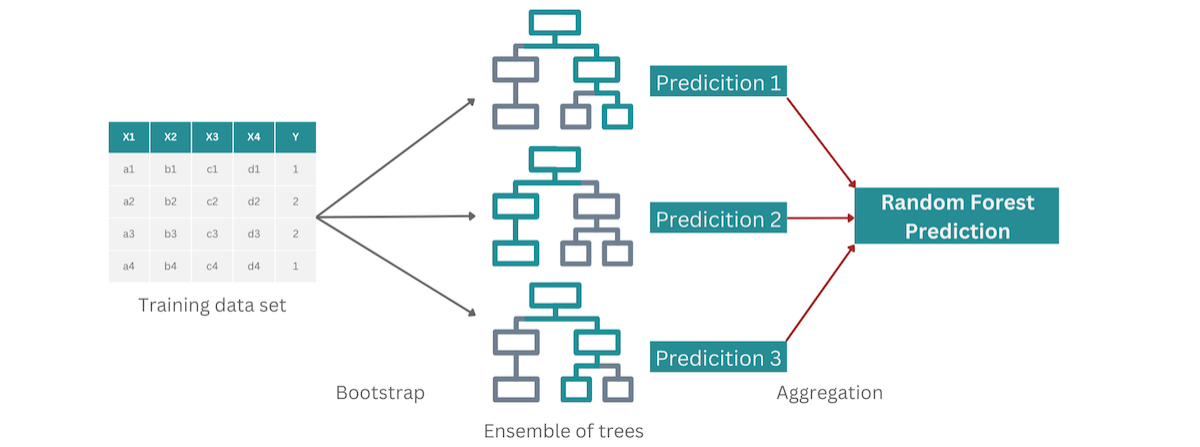
Random forest classification model: a tree-based algorithm [20].

Considering the discrete pain values as distinct classes, 3 classification models were fit to the data: multinomial logistic regression, gradient boosting, and random forest (Figure 1). The machine learning models were trained with half of the data set, and the other half was then used for testing. The performance of these models was compared to 2 basic models, called “null models,” which used no biometric measures in their prediction. The 2 null models, mode and random, predicted pain scores based on the frequency of the scores in the training set. The mode model assumed that the future pain score would be equal to the most common pain score in the data set, whereas the random model assumed the probability of each pain score to be equal to the frequency in which the score appeared in the data set. It should be noted that these null models are of no clinical significance but are used as a comparison to assess the validity and accuracy of our classification models. If the models we created were no better than the null models, there would be no validity in using the created models. As our evaluation metrics, we considered micro-averaged accuracy, micro-averaged *F*_1_-score, area under the receiving operating characteristic curve, and root-mean-square error (RMSE; Textbox 1). Except for RMSE, the higher the metrics, the better the model. We also use cross-validation to further validate the strength of the models. Using cross-validation, we can use all the data to assess the performance of the models through multiple iterations. For the training-testing split to be a good representation of the overall data, including the class imbalance, we use stratified-10-fold cross-validation [19].

**Definitions table of the used metrics to evaluate the performance of each model.**

**Accuracy**

- Refers to the proportion of correct data points predicted by the machine learning algorithm out of all data points.

**Micro-averaging**

- A way to redefine certain statistical measures to deal with class imbalance, where we take the weighted average of the scores for each class. (“1” is considered a perfect model.)

***F*_1_-score**

- Considers not only the accurate recall of a model but also how close together predicted values are to each other. (“1” is considered a perfect model.)

**Area under the receiving operating characteristic curve**

- Determines how well our model picks between different pain score classes. In our model, each numerical pain score is a class. (“1” is considered a perfect model.)

**Root-mean-square error**

- Refers to how far the true values are from values predicted by our model. Larger values represent the further distance between predicted and true values.

### Ethics Approval

The study protocol was approved by the institutional review board of Duke University Medical Center (IRB Pro00068979). All study participants signed consent prior to study participation, and no compensation was provided. Identifiable personal information was not collected in this study, and all data were kept confidential according to the internal data security policy. Data were only accessible to authorized researchers.

## Results

Our study population included 20 patients, including 12 (60%) females. All participants had a confirmed diagnosis of SCD including 14 individuals with hemoglobin type SS (70%), 5 with hemoglobin type SC (25%), and 1 with hemoglobin type SO^Arab^ (5%). All participants identified as Black or African American. A detailed breakdown of the collected data across the sample population is included (Table 1). The median age was 35.5 (IQR 30-41) years. The included patients wore the Apple Watch for the average time of 2 hours 17 minutes.

**Figure 2:**
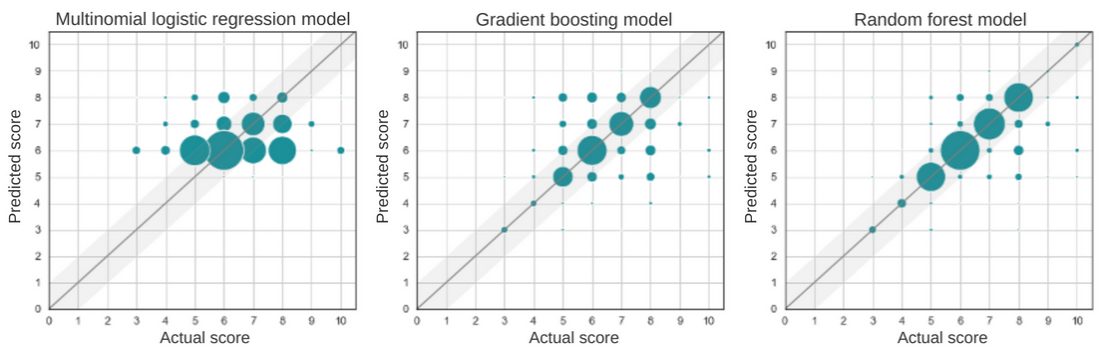
Scatter plots for the 3 models (multinomial logistic regression, gradient boosting, and random forest). The size of the marker represents the number of data points on the corresponding grid point. The straight line along with the shaded region represents the predicted pain score=true pain score±1.

**Figure 3:**
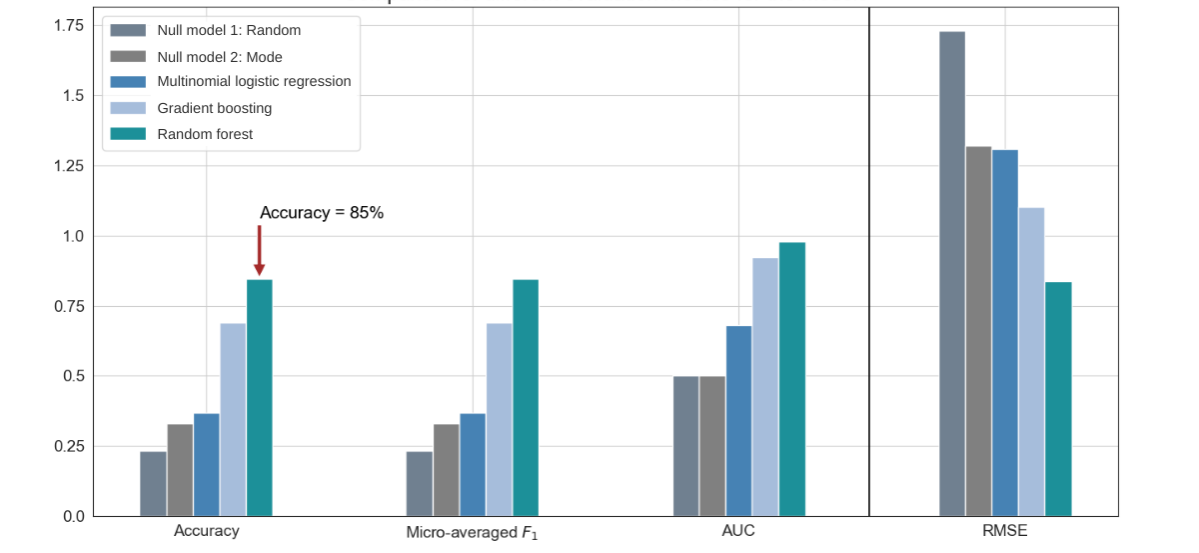
Bar graph comparison of the evaluation metrics for each model along with 2 null models. AUC: area under the receiving operating characteristic curve; RMSE: root-mean-square error.

**Figure 4:**
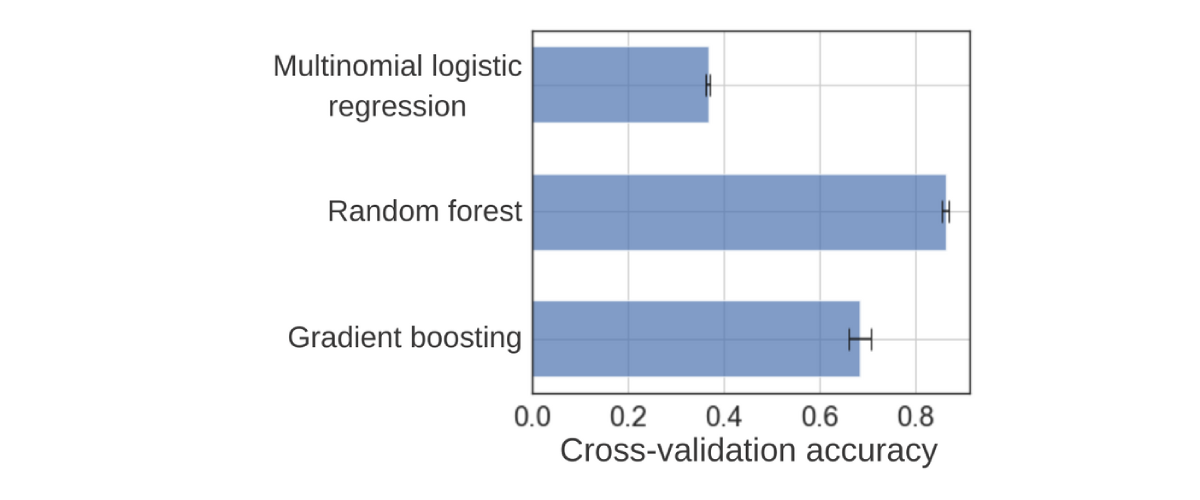
Bar graph comparison of the 10-fold cross-validation accuracy for the 3 machine learning models. The error line is the SD of the accuracies achieved at each fold.

All models outperformed the 2 null models created, and the random forest model significantly outperformed all models followed by the gradient boosting model (Table 2). The scatter plots in Figure 2 show that the model was not able to predict some pain scores (0-2) and that it worked best for certain scores (5-8). This was, respectively, due to the absence of patients reporting very low pain scores, given that the data were collected from patients during a VOC, which created a class imbalance in the data. A comparison of each model is found in Figure 3, and it is evident that even the worst-performing model that uses biometric data, the multinomial logistic regression model, is stronger than the null models. Figure 4 shows the mean and SD of cross-validation accuracy for the 3 models over the 10 folds. We see that the SD for all 3 models is fairly small, indicating that the models are most likely to perform equally well for an independent data set.

**Table 1:** Additional information on the collected data across the sample population.

	Median (IQR)
Number of data points per patient	980 (672 to 1282)
Time spent wearing Apple Watch	2 hours 11 minutes (1 hour 31 minutes to 2 hours 50 minutes)
Age of patients, years	35.5 (30 to 41)
Pain score on entry to Day Hospital	8 (7.5 to 8.5)

**Table 2:** The performance of each model including 2 null models.

Prediction model	Accuracy (%)	Micro-averaged *F*_1_-score	AUC^a^	RMSE^b^
Null model 1: random	23.83	0.24	0.5	1.77
Null model 2: mode	32.92	0.33	0.5	1.32
Multinomial logistic regression fit	37.72	0.37	0.68	1.30
Gradient boosting fit	69.06	0.69	0.92	1.10
Random forest fit	84.52	0.85	0.98	0.84

^a^AUC: area under the receiving operating characteristic curve.

^b^RMSE: root-mean-square error.

## Discussion

### Principal Results

In this study, we were able to show the feasibility of using the Apple Watch Series 3 to collect biometric data during treatment for VOC in adults with sickle cell disease, and that the data collected along with pain scores recorded within EMR could be used to build accurate machine learning models. The random forest model was our best-performing machine learning model, with an accuracy of 84.5%. Considering all the measures, our analyses showed the success of using biometric data collected from a consumer wearable, the Apple Watch, for use in dependable and accurate machine learning pain prediction models.

These results, and those from our previous study with the Microsoft Band 2 [6], continue to be encouraging the potential of consumer wearables in pain prediction for VOCs in individuals with sickle cell disease. In the previous study using the Microsoft Band 2 and a mobile app for data collection, we were able to achieve a pain prediction accuracy of 72.9% using a regression machine learning model. A primary difference in the studies, aside from the device used, was the method of pain score variable collection. This study used only the nurse-recorded pain scores from the EMR, which meant that the pain scores were discrete instead of continuous variables, so classification models were the best fit and resulted in higher accuracy in their pain score prediction. A review of machine learning and pain studies by Matsangidou et al showed the prevalence of this research, reviewing 26 papers published between 2015 and 2021 on pain and machine learning [21]. Within the studies reviewed, many were using machine learning to classify or predict the manifestation of pain in relation to conditions such as osteoarthritis, spinal cord injury, ankylosing spondylitis, and various types of back pain. These studies were all able to predict pain with accuracies above 50% in their best-performing machine learning models, with some achieving accuracies of 90% [21]. The multitude of papers available on using machine learning and pain prediction shows that this is a promising and upcoming field. However, we continue to find a lack of studies, outside our efforts, using the combination of biometric data collected from wearables and machine learning to predict pain in SCD.

### Importance of the Work

Predictive pain tools have the potential to help people living with sickle cell disease, and their medical teams notice trends in their symptoms and pain. There exists a positive correlation between anxiety around pain and pain severity, which can lead to higher pain levels in people with sickle cell disease due to increased anxiety around their expected symptoms [22]. People with sickle cell disease have very high readmission rates associated with excessive costs [23,24]. Treating pain early on could prevent the pain cascade [25], and this could reduce the need for further intervention by medical providers. A tool that both validates their pain and potentially predicts future pain may lower anxiety by giving more information surrounding their standardized pain score, enabling preventative treatments for pain. This may also result in someone living with sickle cell disease coming into the hospital or primary care to receive treatment for pain earlier. Also, SCD primarily affects people of color who have a history of being mistreated or undertreated by the health care system in the United States [26,27]. Prediction models can help validate and support a patient’s own experience and can provide them with a voice in a space where they may have felt like they have less of one. All of the above may result in both individuals with sickle cell disease and hospitals alike saving money regarding treatment, admission, and readmission rates for in-patient care.

### Strengths and Limitations

Our results from the machine learning models are very promising and could significantly improve the treatment of pain from VOCs. A key strength in our methods is that the biometric data collected came from a consumer wearable device, the Apple Watch, and led to accurate predictive machine learning models. This means that data can be collected noninvasively and passively but used to create clinically relevant information.

Overall, 3 classification machine learning models were used to compare and evaluate their ability to predict pain scores with the data set but were also compared to 2 null models. This further strengthens our results, by comparing the trained machine learning models not only to each other but also to null models that used no biometric data. In having all 3 trained models outperform the null models, we provided a check that our prediction results using the trained models were valid. In comparing the 3 trained models, we were able to determine that 1 had the greatest success, the random forest model. Our cross-validation analyses show that our models including the random forest model will perform equally in an independent data set; however, external validation using other data sets is necessary to determine the reproducibility and generalizability of this model for all patients with sickle cell disease living in and outside the United States.

The study included limitations that should be discussed. The participant pool was created via a single-center Day Hospital, which is an option for those who are experiencing high levels of pain but not available in most hospitals. All participants were treated for pain management based on individual pain plans and had high levels of pain upon enrollment into the study. This led to the majority of reported pain scores from participants being within the 5-8 range in the 11-point pain scale (0-10), with no scores reported in the 0-2 range. We used micro-averaging (Textbox 1) to take into account this class imbalance. A full range of reported scores by including data from participants who are not experiencing pain or severe pain will help with the class imbalance. We also have a small sample size of 20 patients, which resulted in less data for the machine learning algorithms. Even with our limited sample size, we were able to create machine learning models that performed well, and better than the null models, in all metrics.

In future research, related to sample size, we plan to expand on our available data set both in patients enrolled and length of time in collecting data from the wearable device, to include time periods both in VOC pain crises as well as outpatient periods when not in significant pain. In collecting large quantities of data from a larger population, we can further train and evaluate our machine learning models for pain prediction and remove the class imbalance seen in this data set. The positive outcomes of the research provide support for the use of consumer wearables in the health care system; still, several difficulties have led to limited adoption [28]. One reason is the number of restrictions around the implementation of these consumer wearables in research studies or clinics. The time and money needed to deploy such consumer wearable initiatives require many financial resources. There also still exists some stigma around the implementation of consumer wearables in clinics [29], even with their Food and Drug Administration approval. However, with the COVID-19 pandemic illuminating the need for various remote monitoring services and other ways of managing the health care of so many people, these tools have become more accepted to manage a wide variety of conditions. Every year, more and more insurance companies are providing billable reimbursement for not only the consumer wearable itself but also reimbursing the provider team for the time spent managing these consumer wearables in the health care system. With these improvements, we hope to see that these tools have greater usage for not only the areas of chronic pain, but also any others.

### Conclusions

Given our results in this study, machine learning can use biometric data from the Apple Watch to become a tool to predict pain scores but will require further validation. Collected information via consumer wearables can be beneficial to patients, clinicians, and hospitals, due to its ability to provide a voice to patients’ symptoms, give clinicians an additional tool for pain reference, and potentially reduce the resource burden on hospitals.

## Abbreviations

EMR: electronic medical record
HR: heart rate
HRV: heart rate variability
RMSE: root-mean-square error
SCD: sickle cell disease
VOC: vaso-occlusive crisis

## Data Availability

The data sets generated and analyzed during this study are available from the corresponding author on reasonable request.
